# Therapeutic Notes

**Published:** 1894-12

**Authors:** 


### Therapeutic Notes.

Dr. Seneca D. Powell, professor of clinical surgery at the New York Post-Graduate Medical School and Hospital, at a recent clinic, in speaking of the treatment of inherited syphilis, said:

The Maltine Manufacturing Company have recently added another valuable preparation to their list, maltine with coca wine. It is especially indicated in cases where the general condition is below par and a tonic without too much alcohol. I have also been using it in old people who are not especially ill, but are feeble and debilitated in the afternoon. It seems to pick them up and is not followed by the usual reaction of alcoholic stimulants. You will find a fruitful field for its use in children of low vitality, who are poor starch digestors, and in convalescence from wasting diseases and exhaustive operations.

---

Boiled Water for School Children.—The prefects in the several French departments have issued orders to the various schools, requiring that all drinking water supplied to the pupils shall be boiled, and that the cleansing of the floors, desks, etc., of the school rooms be no longer done with dry dusters and brooms, but with moist cloths, to prevent spread of dust. Once a week a thorough cleansing is to be carried out with an antiseptic. —*Memphis Medical Monthly.*

